# Intracellular Behaviour of Three *Legionella pneumophila* Strains within Three Amoeba Strains, Including *Willaertia magna* C2c Maky

**DOI:** 10.3390/pathogens9020105

**Published:** 2020-02-06

**Authors:** Issam Hasni, Antoine Jarry, Benjamin Quelard, Antoine Carlino, Jean-Baptiste Eberst, Olivier Abbe, Sandrine Demanèche

**Affiliations:** 1 Microbes, Evolution, Phylogeny and Infection Department, Institut de Recherche pour le Développement IRD 198, Institut Hospitalo-Universitaire (IHU), Aix-Marseille Université, 13007 Marseille, France; issam.hasni@amoeba-biocide.com; 2 R&D Department, Amoéba, 38 Avenue des Frères Montgolfier, 69680 Chassieu, France; antoinejarry93@gmail.com (A.J.); benjamin.quelard@amoeba-biocide.com (B.Q.); Antoine.CARLINO@amoeba-biocide.com (A.C.); Jean-Baptiste.EBERST@amoeba-biocide.com (J.-B.E.); Olivier.Abbe@amoeba-biocide.com (O.A.)

**Keywords:** free-living amoebae, *Legionella*, biological biocide, cooling towers

## Abstract

*Legionella pneumophila* is a facultative intracellular pathogen found in aquatic environments as planktonic cells within biofilms and as intracellular parasites of free-living amoebae such as *Acanthamoeba castellanii*. This pathogen bypasses the elimination mechanism to replicate within amoebae; however, not all amoeba species support the growth of *L. pneumophila*. *Willaertia magna* C2c Maky, a non-pathogenic amoeba, was previously demonstrated to possess the ability to eliminate the *L. pneumophila* strain Paris. Here, we study the intracellular behaviour of three *L. pneumophila* strains (Paris, Philadelphia, and Lens) within *W. magna* C2c Maky and compare this strain to *A. castellanii* and *W. magna* Z503, which are used as controls. We observe the intracellular growth of strain Lens within *W. magna* Z503 and *A. castellanii* at 22 °C and 37 °C. Strain Paris grows within *A. castellanii* at any temperature, while it only grows at 22 °C within *W. magna* Z503. Strain Philadelphia proliferates only within *A. castellanii* at 37 °C. Within *W. magna* C2c Maky, none of the three legionella strains exhibit intracellular growth. Additionally, the ability of *W. magna* C2c Maky to decrease the number of internalized *L. pneumophila* is confirmed. These results support the idea that *W. magna* C2c Maky possesses unique behaviour in regard to *L. pneumophila* strains.

## 1. Introduction

*Legionella pneumophila* is an aerobic, Gram-negative bacterium that causes Legionellosis, a severe form of pneumonia, following inoculation with contaminated aerosol [1]. This bacterial infection manifests as two clinical forms that include Legionnaires’ disease, which is a life-threatening respiratory disease, and Pontiac fever, a milder self-limiting illness [2,3]. Among the sixteen currently identified serogroups of *L. pneumophila*, serogroup 1 is involved in the majority of infections [4,5]. This microorganism is ubiquitous throughout natural and artificial aquatic environments [6]. Legionellosis outbreaks are frequently related to contaminated water systems that produce aerosols, which occurs primarily within cooling towers [7]. Indeed, cooling towers provide ideal conditions for pathogen growth, as they frequently possess temperatures above 20 °C, at which *L. pneumophila* can proliferate [8,9,10].

Free-living amoebae (FLA) are ubiquitous protozoa that inhabit common aquatic environments and are frequently co-isolated with *L. pneumophila* in water cooling towers [11,12]. FLA are predatory and consume bacteria to facilitate their growth [13,14]; however, some bacteria such as *L. pneumophila* have evolved to avoid the phagolysosome fusion and can multiply within FLA, ultimately killing these amoebae before disseminating into the environment [9,15,16,17]. Furthermore, amoeba cysts can provide *L. pneumophila* with protection against unfavourable conditions and chemical treatments. Therefore, the association between FLA and this pathogen makes the control and monitoring of water-cooling towers difficult and makes eradication of *L. pneumophila* almost impossible [18,19].

Previous studies, however, have demonstrated that all FLAs do not exhibit the same behaviours when they come into contact with *L. pneumophila* strains. While *Acanthamoeba* sp. and *Vermamoeba* (formerly *Hartmannella*) *vermiformis* support the intracellular growth of *L. pneumophila,* the *Willaertia magna* strain C2c Maky has been demonstrated to eliminate the *L. pneumophila* serogroup 1 strain Paris ATCC 33152 [20], which is a virulent pathogen strain responsible for severe legionellosis epidemics in France [21]. *W. magna* C2c Maky is a free-living amoeba that is a member of the Vahlkampfiidae family [22]. This amoeba is a thermophilic FLA that is isolated from the water of thermal swimming pools (http://www.amoeba-biocide.com/en/page/learn-more-about-willaertia-magna-c2c-maky), and it has the capacity to grow at high temperatures (up to 44 °C) in xenic or axenic media. The living forms of this amoeba include a large trophozoite (50–100 µm) and a cyst (18–21 µm) form, and it can produce temporary flagella [22,23]. The lack of pathogenicity of this amoeba was demonstrated by cytotoxicity testing on human cells and was confirmed by genomic analysis [24]. According to these findings, the Amoéba company developed a natural biocide using *W. magna* C2c Maky to eliminate *L. pneumophila* as an alternative to chemical biocides (http://www.amoeba-biocide.com/en/page/revolutionary-biocide). The present study is performed to verify the elimination and the absence of the reservoir effect. Specifically, the decrease in the number of internal *L. pneumophila* and the absence of internal *L. pneumophila* multiplication within *W. magna* C2c Maky, when both microorganisms are co-cultured, is confirmed. The assay is performed by examining adhesion (the usual way of life for free-living amoeba) with three strains of *L. pneumophila* to assess the consistency of amoeba behaviour toward legionella strains. The assay lasts for one week and includes a daily count of intracellular *L. pneumophila* and amoebas by culture and Trypan blue staining, respectively. The behaviour of *W. magna* C2c Maky is compared to that of *W. magna* Z503 to determine if two amoeba strains of the same species have the same behavior. Moreover, it is compared to *A. castellanii*, an amoeba known to multiply amoeba-resistant bacteria such as the three *L. pneumophila* strains studied. 

## 2. Results

### 2.1. L. pneumophila Survival in Coculture Medium

**Figure 1:**
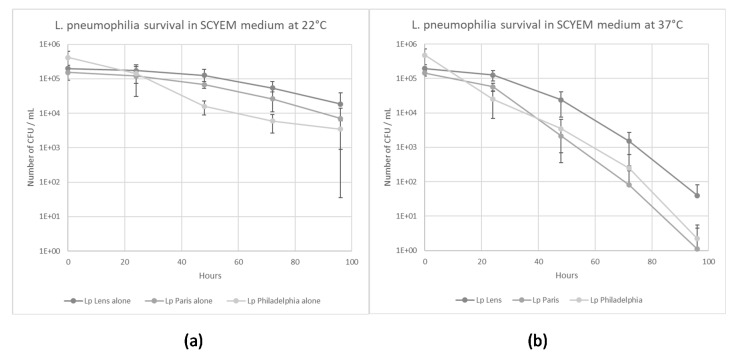
*L. pneumophila* survival in coculture medium at 22 °C (**a**) and 37 °C (**b**). Results are expressed as the mean +/− 95% CI (Confidence Interval based on the standard error of the mean).

The survival of the three *L. pneumophila* strains in the calf serum-casein-yeast extract medium (SCYEM) was evaluated at 22 °C and 37 °C (Figure 1a,b). The survival of *L. pneumophila* Lens decreased to 2 × 10^4^ CFU/mL and to 40 CFU/mL in SCYEM medium within 96 h at 22 °C and 37 °C, respectively. The survival of *L. pneumophila* Paris decreased to 7 × 10^3^ CFU/mL and to 1 CFU/mL in SCYEM medium within 96 h at 22 °C and 37 °C, respectively. Finally, the survival of *L. pneumophila* Philadelphia decreased to 3 × 10^3^ CFU/mL and to 2 CFU/mL in SCYEM medium within 96 h at 22 °C and 37 °C, respectively.

### 2.2. Amoeba Survival in coculture Medium

**Figure 2:**
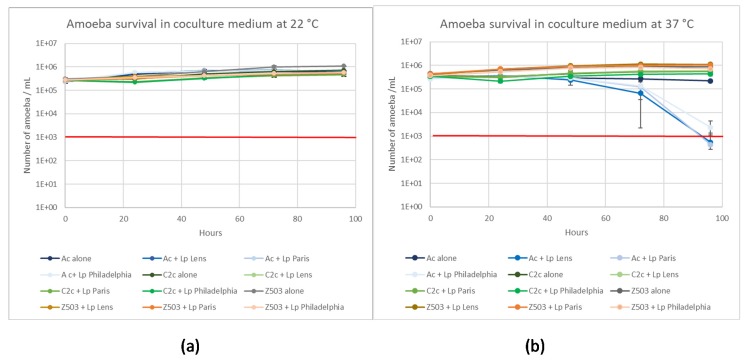
Amoeba survival at 22 °C (**a**) and 37 °C (**b**) in coculture medium in the presence or absence of the three *L. pneumophila* strains (Lens, Paris, and Philadelphia). The red bar is the detection limit of the Malassez cell counting. Results are expressed as the mean +/− 95% CI (Confidence Interval based on the standard error of the mean).

Survival of the three amoebas in the presence or in absence of bacteria was evaluated over 96 h at 22 °C and 37 °C in coculture medium (Figure 2a,b). The three amoeba strains could be maintained in SCYEM medium for 96 h in the presence or absence of bacteria at 22 °C and 37 °C with the exception of *A. castellanii* when co-cultivated with *L. pneumophila* strains. Found at the end of the experiment, the control condition of *A. castellanii* in the absence of bacteria was maintained at 2 × 10^5^ cells/mL, while in the presence of *L. pneumophila* Lens, Paris, and Philadelphia, the amoeba number decreased to 556, 444 and 2333 cells/mL, respectively (Figure 2b). *A. castellanii* could not survive in the presence of the three *L. pneumophila* strains at 37 °C.

### 2.3. Co-Culture Experiments 

#### 2.3.1. *L. pneumophila* Lens co-cultivated with Amoeba Strains

The mean initial amount of amoeba-internalized bacteria at 22 °C was 16 ± 0.5% (16% in *A. castellanii*, 15% in *W. magna* C2c Maky, and 16% in *W. magna* Z503). Seen at 37 °C, a mean bacterial uptake of 20 ± 5.5% was observed (15% in *A. castellanii*, 26% in *W. magna* C2c Maky, and 18% in *W. magna* Z503). 

**Figure 3:**
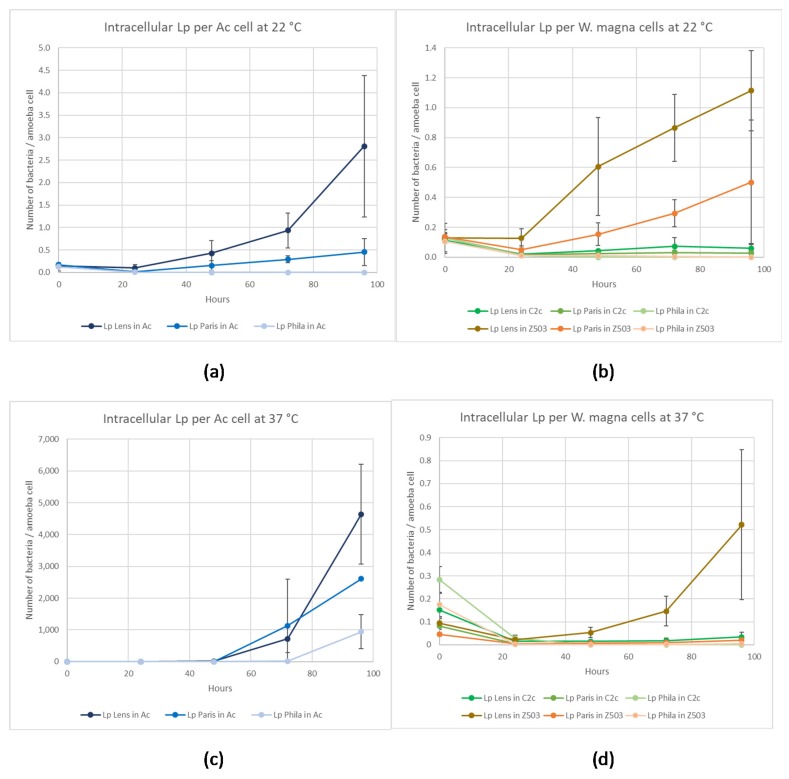
Comparison of the evolution of the number of intracellular *L. pneumophila* cells (Lens, Paris, and Philadelphia) per amoeba cell (*A. castellanii, W. magna* C2c Maky, and *W. magna* Z503). Results are expressed as the mean +/− 95% CI (Confidence Interval based on the standard error of the mean). (**a**) *L. pneumophila* number per *A. castellanii* cell at 22 °C (n = 9 for Lp Lens and Paris, n = 15 for Lp Philadelphia); (**b**) *L. pneumophila* number per *A. castellanii* cell at 37 °C (n = 9); (**c**) *L. pneumophila* number per *W. magna* cell (C2c and Z503) at 22 °C (n = 9 for Lp Lens and Paris, n = 15 for Lp Philadelphia); (**d**) *L. pneumophila* number per *W. magna* cell (C2c and Z503) at 37 °C (n = 9).

A significant decrease (*p* < 0.05) in the number of intracellular *L. pneumophila* Lens per *W. magna* C2c Maky cell was observed after 24 h (5-fold and 10-fold reduction at 22 °C and 37 °C, respectively), while the level remained nearly constant for *A. castellanii* at 22 °C and 37 °C and for *W. magna* Z503 at 22 °C with no significant difference between T_0_ and T_0_ + 24 h (*p* > 0.05) (Figure 3). Occurring at T_0_ + 96 h (Figure 3), the percentage of intracellular *L. pneumophila* Lens per *W. magna* C2c Maky cell was reduced by 48 ± 0.3% at 22 °C and 77 ± 1.2% at 37 °C, and an increase was observed for *W. magna* Z503 (9-fold at 22 °C and 5-fold at 37 °C) and *A. castellanii* (19-fold at 22 °C and 50,000-fold at 37 °C). Observed at 37 °C, a small number of *A. castellanii* cells were still alive (5.6 × 10^2^ ± 5.9 × 10^2^ amoebas/mL), demonstrating that amoeba cell lysis occurred following the intracellular multiplication of *L. pneumophila* Lens.

**Figure 4:**
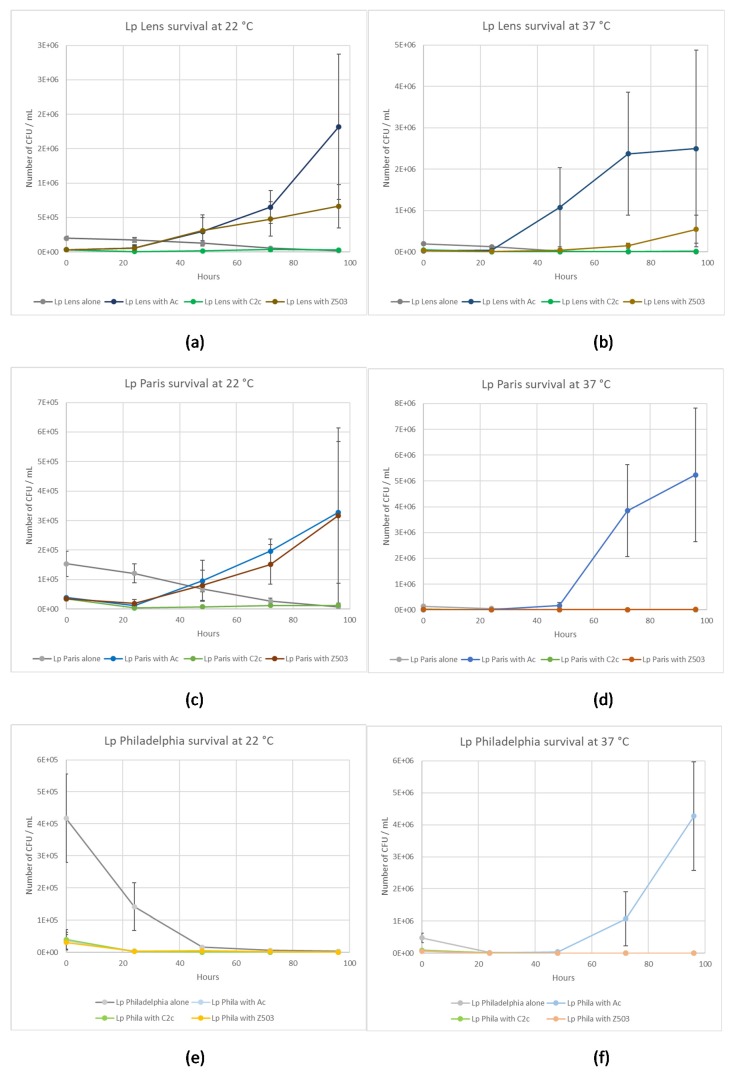
Comparison of the evolution of the number of *L. pneumophila* cells in the presence or absence of amoeba cells (alone, or in presence of *A. castellanii*, *W. magna* C2c Maky, or *W. magna* Z503). Results are expressed as the mean +/− 95% CI (Confidence Interval based on the standard error of the mean). (**a**) *L. pneumophila* Lens at 22 °C (n = 9); (**b**) *L. pneumophila* Lens at 37 °C (n = 9); (**c**) *L. pneumophila* Paris at 22 °C (n = 9); (**d**) *L. pneumophila* Paris at 37 °C (n = 9); (**e**) *L. pneumophila* Philadelphia at 22 °C (n = 15); (**f**) *L. pneumophila* Philadelphia at 37 °C (n = 9).

Considering the number of *L. pneumophila* Lens at 22 °C and 37 °C, a significant increase (*p* < 0.05) was obtained when the bacterium was co-cultivated with *W. magna* Z503 and *A. castellanii*, and this was not observed when *L. pneumophila* Lens was cultivated alone or in the presence of *W. magna* C2c Maky (Figure 4a,b), demonstrating an intracellular multiplication of *L. pneumophila* Lens in *W. magna* Z503 and *A. castellanii* as the bacterium was unable to multiply by itself in the coculture medium (Figure 1a,b). 

#### 2.3.2. *L. pneumophila* Paris Co-Cultivated with Amoeba Strains

Occurring at 22 °C, we reported a mean *L. pneumophila* Paris uptake by amoebas of 24 ± 1.5% (25% in *A. castellanii*, 23% in *W. magna* C2c Maky, and 23% in *W. magna* Z503). The initial mean amount of cells internalized by amoebas decreased to 14 ± 5.0% at 37 °C (9% in *A. castellanii*, 19% in *W. magna* C2c Maky and 13% in *W. magna* Z503). 

A significant decrease of the number of intracellular *L. pneumophila* Paris per amoeba cell (*p* < 0.05) first was observed in the three amoebas after 24 h, with the exception of *A. castellanii* at 37 °C (8-fold for *W. magna* C2c Maky, 3-fold for *W. magna* Z503, and 9-fold for *A. castellanii* at 22 °C and 19-fold for *W. magna* C2c Maky, 11-fold for *W. magna* Z503, and 2-fold for *A. castellanii* at 37 °C) (Figure 3). This decrease was maintained until the end of the experiment (T_0_ + 96 h) only by *W. magna* C2c Maky, and the percentage of intracellular *L. pneumophila* Paris per amoeba cell was reduced by 79 ± 2% at 22 °C and 98 ± 0.1% at 37 °C (*p* < 0.05). The opposite was observed for *W. magna* Z503 and *A. castellanii* at 22 °C and 37 °C, as the decrease measured after 24 h was not maintained. Seen at 48 h, the level of intracellular *L. pneumophila* Paris per amoeba cell began to increase until it reached 4-fold and 3-fold more bacteria per amoeba cell than that observed at T_0_ for *W. magna* Z503 and *A. castellanii*, respectively at 22 °C. Observed at 37 °C for *W. magna* Z503, the number of intracellular *L. pneumophila* Paris per amoeba cell at T_0_ + 96 h was 5-fold the ratio observed at 24 h, but it did not reach the initial ratio. Regarding *A. castellanii*, a strong increase was observed at both temperatures, and the initial ratio was slightly increased by 3-fold at 22 °C (*p* > 0.05) and strongly increased by 60,000-fold at 37 °C (*p* < 0.05). Furthermore, the correlation between the increase in *L. pneumophila* Paris and the low concentration of viable *A. castellanii* (5.6 × 10^2^ ± 5.9 × 10^2^ cells/mL) after 96 h indicated that a high intracellular multiplication of *L. pneumophila* Paris occurred that was followed by a release of bacteria in the medium after *A. castellanii* death.

Considering the number of *L. pneumophila* Paris at 22 °C, a significant increase (*p* < 0.05) was obtained when the bacterium was co-cultured with *W. magna* Z503 and *A. castellanii*, and this was not observed when *L. pneumophila* Paris was cultured alone or in the presence of *W. magna* Z503 at 37 °C and *W. magna* C2c Maky at both 22 °C and 37 °C (Figure 4c,d), demonstrating an intracellular multiplication of *L. pneumophila* Paris in *W. magna* Z503 and *A. castellanii* at 22 °C and only in *A. castellanii* at 37 °C as the bacterium was unable to multiply by itself in the coculture medium (Figure 1a,b). 

#### 2.3.3. *L. pneumophila* Philadelphia Co-Cultivated with Amoeba Strains

The mean bacterial internalization by amoebas was 9 ± 1.1% (9% in *A. castellanii,* 10% in *W. magna* C2c Maky, and 7% in *W. magna* Z503) at 22 °C, and the initial amount of internalized cells by amoebas increased to 17 ± 3.8% (19% in *A. castellanii*, 20% in *W. magna* C2c Maky, and 13% in *W. magna* Z503). 

Occurring at 22 °C, a rapid and significant (*p* < 0.05) decrease in the number of intracellular *L. pneumophila* per amoeba cell was observed within 24 h (20-fold for *A. castellanii*, 11-fold for *W. magna* C2c Maky, and 10-fold for *W. magna* Z503) in the three amoebas (Figure 3). Then, a slow but significant (*p* < 0.05) decrease continued until the death of more than 99% of intracellular *L. pneumophila* Philadelphia in all cases. Even if this decrease could be attributed to the bacterial death in the coculture medium, the experiment demonstrated the absence of intra-amoeba multiplication of *L. pneumophila* Philadelphia necessary for survival at 22 °C. 

Occurring at 37 °C, a similar rapid decrease in the number of intracellular *L. pneumophila* per amoeba was observed within 24 h for all three amoebas (20-fold for *A. castellanii*, 10-fold for *W. magna* C2c Maky, and 92-fold for *W. magna* Z503). Then, differential behaviours were observed depending on the amoeba strains. Regarding *W. magna* C2c Maky, the significant decrease (*p* < 0.05) continued until the death of more than 99.99% of the intracellular *L. pneumophila* Philadelphia per amoeba cell (Figure 3d). Concerning *W. magna* Z503, a decrease also was observed up to 97% elimination of intracellular *L. pneumophila* Philadelphia per amoeba cell after 96 h (*p* < 0.05) (Figure 3d). To contrast, for *A. castellanii*, a significant increase (*p* < 0.05) in intracellular *L. pneumophila* Philadelphia per amoeba cell appeared after 48 h, demonstrating an intra-amoeba multiplication up to 2600-fold at the end point (Figure 3c).

Considering the number of *L. pneumophila* Philadelphia at 22 °C, a significant decrease (*p* < 0.05) was obtained in all cases (Figure 4e), while at 37 °C, a significant increase (*p* < 0.05) was observed when *L. pneumophila* Philadelphia was cultured in the presence of *A. castellanii* (Figure 4f). This demonstrated an intracellular multiplication of *L. pneumophila* Philadelphia *A. castellanii* at 37 °C, as the bacterium was unable to multiply by itself in SCYEM medium (Figure 1a,b). 

### 2.4. Microscopic Observations of Intracellular L. pneumophila Philadelphia at 37 °C

**Figure 5:**
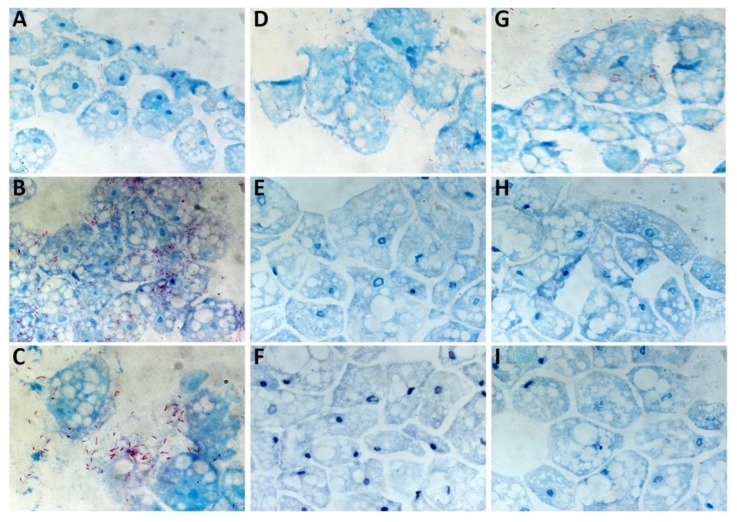
Optical microscopy observation using Gimenez staining of *A. castellanii* (**A**–**C**), *W. magna* C2c Maky (**D**–**F**), and *W. magna* Z503 (**G**–**I**) infected with *L. pneumophila* Philadelphia at 37 °C. Photos of the co-cultures were acquired at T_0_ (**A**,**D**,**G**), T_0_ + 48 h (**B**,**E**,**H**), and T_0_ + 96 h (**C**,**F**,**I**).

Microscopic observations were performed at T_0,_ T_0_ + 48 h, and T_0_ + 96 h. Occurring at T_0,_ excess intracellular *L. pneumophila* Philadelphia bacteria were observed in the presence of the three amoebas (Figure 5A,D,G). Regarding *A. castellanii* at 48 h, a strong bacterial multiplication was observed (Figure 5B) which was not observed for both *W. magna* strains (Figure 5E,H). Occurring at 96 h, lysis of *A. castellanii* after intracellular bacterial multiplication was clearly evident (Figure 5C), and only a small amount of amoeba lysis could be observed for both *W. magna* strains (Figure 5F,I). 

### 2.5. Statistical Comparison of Amoeba Behavior

Analysis of variance tests (ANOVA) were performed to determine if *W. magna* C2c Maky interacted with *L. pneumophila* in a significantly different manner compared to interactions with the two other amoebas. 

Concerning the three bacterial strains, T_0_ data obtained in the presence of the three amoebas were not statistically different at 22 °C (*p* > 0.05); however, at 37 °C, a significant difference in behaviour (*p* < 0.05) was detected at T_0_. 

Pairwise comparisons (Dunn test) established that at 72 h and 96 h at both temperatures and with the three legionella strains, *W. magna* C2c Maky behaviour was statistically different from that of the two other amoeba strains (Table 1). This significant difference was observed even after 24 h with strain Paris at both temperatures, and at 22 °C for strain Lens. Statistical tests provided evidence that *W. magna* C2c Maky behaved differently compared to *W. magna* Z503 and *A. castellanii* cells in the presence of *Legionella* strains.

## 3. Discussion

This work explores the permissiveness of three amoeba strains regarding the intracellular multiplication of three pathogenic *L. pneumophila* strains under two temperature conditions (22 °C and 37 °C) that correspond to temperatures found in cooling towers in which *L. pneumophila* are known to replicate within certain strains of amoebae [10,25]. It is important to demonstrate that *W. magna* C2c Maky does not multiply *L. pneumophila* as we aim to propose it as a natural biocide to treat cooling towers.

The three *L. pneumophila* strains are a representative set of *L. pneumophila* serogroup 1 that is responsible for 95% of the legionellosis disease world-wide [5]. Strain Philadelphia is a clinical isolate that is historically responsible for the very first outbreak. It possesses gene traits that allow for multiplication in a number of hosts such as peripheral blood mononuclear cells, peritoneal macrophages, and *A. castellanii*, *A. polyphaga*, or *A. lenticulate* [26,27,28,29]. The Philadelphia strain is, according to the EN 13623 European standard, the only strain for which testing is required to validate a disinfectant against *Legionella* in Europe. *L. pneumophila* Lens was chosen because it was responsible for an outbreak in the north of France between November 2003 and January 2004 where 86 confirmed cases resulted in 17 deaths [30]. *L. pneumophila* Paris was chosen because, among the endemic strains of *L. pneumophila* serogroup 1, sequence type 1 (ST1) strains are among the most prevalent, particularly the ST1/Paris pulsotype. This endemic type was responsible for 8.2% of French culture-proven cases of Legionnaire’s disease from 1995 through 2006. ST1/Paris pulsotype isolates also have been detected in clinical and environmental samples taken from several other countries around the world, including Switzerland, Italy, Spain, Sweden, the United States, Japan, Senegal, and Canada [21,30].

Our experiments demonstrate differential behaviours among amoeba species infected by the pathogenic bacteria. Compared to *A. castellanii* and *W. magna* Z503, the intracellular *L. pneumophila* are efficiently eliminated by *W. magna* C2c Maky at 22 °C and 37 °C. Indeed, the experiments report not only a non-replication, but also an elimination of the intracellular strains Lens, Paris and Philadelphia within *W. magna* C2c Maky. Furthermore, the coculture medium used in the survey is not adapted to the survival of the legionella bacteria, and they, therefore, must parasitize the amoebae to facilitate their own growth. Indeed, the experiments demonstrate that the three legionella strains were unable to remain at the inoculation level and began to die after 24 h (Figure 1). Although the medium is not adapted to *L. pneumophila* strains, it was chosen for the co-culture study because an increase of the bacterial number during the co-culture experiment necessarily indicates that the multiplication occurred within amoeba. The bacterial multiplication is observed both in *A. castellanii* and *W. magna* Z503, and it is not observed in *W. magna* C2c Maky. The assays reveal a multiplication of all legionella strains within *A. castellanii* at 37 °C and the intracellular multiplication of strain Lens and Paris at 22 °C. Indeed, the strain Philadelphia grows at 37 °C (Figure 3c) and does not multiply at 22 °C (Figure 3a) within *A. castellanii*. Based on this, these results suggest a behaviour that is influenced by the temperature conditions. Several previous studies revealed the effect of temperature on the relationship between *L. pneumophila* and free-living amoeba (FLA) [9,31,32]. *L. pneumophila* serogroup 1, for example, replicated in *A. castellanii* at 25 °C but were digested at temperatures below 20 °C [25]. Dupuy et al. assessed the ability of 12 amoeba strains of *Naegleria* sp., *Acanthamoeba* sp., and *Vermamoeba* sp. to support the multiplication of *L. pneumophila* Lens at various temperatures (25 °C, 30 °C and 40 °C), and they revealed a more efficient intracellular proliferation with increasing temperatures [33]. Additionally, we did not observe the same behaviour according to the different bacteria and amoeba strains used during our experiments. Indeed, the strain Lens replicates at 37 °C within *W. magna* strain Z503, but not in *W. magna* C2c Maky (Figure 3d). The co-culture at 22 °C of *W. magna* Z503 with *L. pneumophila* strain Paris and strain Lens reveals a multiplication of the bacteria; however, no replication is observed during co-culture with strain Philadelphia (Figure 3b). The difference in amoeba permissiveness has been highlighted previously, especially in regard to *Naegleria*, *Acanthamoeba*, *Vermamoeba* and *Micriamoeba tesseris* [9,34]. The non-replication of legionella within *W. magna* C2c Maky was previously observed with strain Paris [20]. Our study confirms this result, as the resistance of *W. magna* C2c Maky towards *L. pneumophila* Paris is illustrated by the observed significant decrease in the bacterial concentration after 4 days of co-culture at 22 °C and 37 °C (Figure 4c,d). Dey et al. [20], however, reported a moderate increase in strains Philadelphia and Lens within *W. magna* C2c at 37 °C while in our study the intracellular bacterial concentration significantly decreased in culture with *W. magna* C2c Maky at 22 °C and 37 °C. These differences can be explained by the protocol parameters used in the former study, particularly regarding the culture medium and elimination of extracellular bacteria. The authors used serum casein glucose yeast extract medium (SCGYEM) that was favourable to *L. pneumophila* survival, so bacteria were not forced to multiply into amoeba to survive. Additionally, Dey and co-workers did not eliminate extracellular bacteria by centrifugation, and the observed increase could be due to extracellular bacterial replication, such as that resulting from necrotrophic growth as previously demonstrated [35].

*W. magna C2c* Maky is demonstrated to possess a high efficiency for digesting the intracellular *L. pneumophila* cells in all strains used in this survey. The growth of *L. pneumophila* within amoebas is known to enhance the pathogenicity and invasion of *L. pneumophila* [15,36]; however, no intracellular bacterial replication is observed when we infect *W. magna* C2c Maky with *L. pneumophila* strains derived from a first co-culture that was thought to be more virulent (unpublished data).

The action on different *L. pneumophila* strains and the absence of internal proliferation support the fact that *W. magna* C2c Maky could be used as a biocide to combat *L. pneumophila* proliferation in cooling tower water. This observation is consistent with the control of legionella by *W. magna* C2c Maky observed in real conditions during field trials in functioning cooling towers (http://www.amoeba-biocide.com/sites/default/files/180711_cp_amoeba_us_positive_efficacy_field_test_en_vedf_0.pdf). The traditional method to control bacterial growth in cooling tower water is primarily based on the use of chemical biocides [37,38]. Indeed, the oxidizing agent chlorine is the most used product for cooling tower treatment [39]. The chemical biocide is efficient to prevent *L. pneumophila* proliferation, although some previous studies reported incomplete eradication of legionella from installations and progressive re-colonization within these systems within weeks or months [40,41]. Moreover, these chemical biocides are dangerous to the environment, they degrade the installation systems, and they require the application of other products such as anti-corrosive agents [42,43]. Described by Iervolino, treatment with another oxidizing agent (H_2_O_2_/Ag) was inadequate for legionella control, and, instead, it caused a rapid increase of one logarithmic unit [44]. Chemical biocide action also is not completely efficient against biofilms and amoeba cysts that can provide protection against disinfection treatment [16,17,45]. Finally, chemical biocides used in cooling towers can select *L. pneumophila* populations, and chemical biocides can promote resistance to biocides and to human health antibiotics [46,47]. 

To conclude, *W. magna* C2c Maky is not associated with any human or animal infection, and this is in agreement with the lack of pathogenicity demonstrated in vivo and suggested by genomic analysis [24,48]. This organism is likely a safe and efficient candidate for legionella control in cooling towers and could provide an alternative solution to chemical biocides. 

## 4. Materials and Methods 

### 4.1. Free-Living Amoebae Culture 

*Willaertia magna* C2c Maky (ATCC^®^ PTA-7824), *Willaertia magna* Z503 (ATCC^®^ 50035), and *Acanthamoeba castellanii* (ATCC^®^ 30010) were purchased from ATCC and cultivated according to their recommendation into 10 mL of modified PYNFH medium (ATCC medium 1034) in a T-25 tissue culture flask. Amoebae were then grown in cell factories in serum casein yeast extract medium (SCYEM) at 30 °C. SCYEM medium is derived from serum casein glucose yeast extract medium (SCGYEM) medium [49] and contained 10 g·L^−1^ casein, 5 g·L^−1^ yeast extract, 10% foetal calf Serum, 1.325 g·L^−1^ Na_2_HPO_4_, and 0.8 g·L^−1^ KH_2_PO_4_. After 72 h (during exponential phase), the cell factories were gently shaken, and the amoeba suspensions were transferred to 50 mL Falcon^®^ tubes. Amoeba populations were then quantified using a Malassez haemocytometer cell counting chamber method (Thermo Fisher Scientific, France) with Trypan blue by mixing 100 µL of Trypan blue with 100 µL of amoeba sample. According to the results, the amoebae concentration in Falcon^®^ tubes was then adjusted to 3 × 10^5^ cells/mL by the addition of SCYEM. The amoebas were then washed twice in SCYEM using centrifugation at 3000× *g* for 10 min, and the supernatants were then discarded. Amoeba populations were then re-quantified, and the amoeba suspensions were finally adjusted to 3 × 10^5^ cells/mL in 100 mL of SCYEM. A final quantification was performed to verify the concentration. 

Each final solution of *W. magna* C2c Maky, *W. magna* Z503, and *A. castellanii* corresponded to working suspensions that were named AWS_C2C_, AWS_Z503_, and AWS_AC_, respectively (Table 2).

### 4.2. Legionella Pneumophila Cultures 

*L. pneumophila* strain Philadelphia (ATCC 33152), *L. pneumophila* strain Lens (CIP 108280), and *L. pneumophila* strain Paris (CIP 107629) were grown on buffered charcoal yeast extract (BCYE) agar plates (Thermo Fisher Scientific, Dardilly, France) at 36 °C for 72 hours and then harvested by scraping, suspended in phosphate-buffered saline (PBS), centrifuged at 9500 xg for 10 min, and washed once in PBS. The supernatants were then discarded. The *L. pneumophila* suspensions were then diluted in PBS to obtain 3 × 10^7^ bacteria/mL. 

The legionella final suspensions represented the bacterial stock working suspensions, and they were identified as BWS_Phila,_ BWS_Paris_, and BWS_Lens_ (Table 2).

### 4.3. Bacterial Survival in the Coculture Medium (Control)

The three control bacterial conditions were prepared as described in Table 2 by adding 10 mL of SCYEM to the 0.1 mL bacteria working solutions (BWS_Phila,_ BWS_Paris_, or BWS_Lens_) in 25 cm^3^ flasks (Dutscher, Brumath, France) and incubated at 22 °C or 37 °C. This operation corresponded to the T_0_ time point of the bacterial controls. Occurring at T_0,_ T_0_ + 24 h, T_0_ + 48 h, T_0_ + 72 h, and T_0_ + 96 h, 1 mL was sampled in each flask and then serially 10-fold diluted in SCYEM and plated on buffered charcoal yeast extract plates (BCYE) in triplicate. BCYE plates were incubated at 36 °C, and colony forming units (CFU) were counted after 5 days. Each condition was performed for three independent replicates and repeated three times (n = 9).

### 4.4. Amoeba Survival in the coculture Medium (Control)

The three amoeba working solutions (AWS_C2C_, AWS_Z503_, or AWS_AC_) were prepared as described in Table 2 (10 mL of working solutions) and incubated at 22 °C or 37 °C in 25 cm^3^ flasks. Occurring at T_0,_ T_0_ + 24 h, T_0_ + 48 h, T_0_ + 72 h, and T_0_ + 96 h, the flasks were gently shaken, and the numbers of amoeba cells were quantified using a haemocytometer cell counting chamber method with Trypan blue. Each condition was performed for three independent replicates and repeated three times (n = 9).

### 4.5. Co-Culture Assays

Amoeba and bacterial working solutions were mixed in 25 cm^3^ flasks by adding the required volume according to Table 1. To provide an example, 10 mL of *W. magna* C2c Maky at 3 × 10^5^ cells/mL was mixed with 0.1 mL of *L. pneumophila* Lens at 3 × 10^7^ CFU / mL. All flasks were left to stand for 2 h at 22 °C ± 2 °C or at 37 °C ± 2 °C to allow for amoebae/bacteria contact and the internalization of *L. pneumophila* into amoebae. After the 2-h contact process, each flask was gently shaken 10 times, and the suspension was transferred into a 15 mL Flacon^®^ tube and centrifuged at 3000× *g* for 5 min. This step allowed for the removal of non-internalized (i.e., extracellular) *L. pneumophila* from the co-culture suspensions. The pellet was resuspended in 10 mL of sterile SCYEM, and the suspension was poured into a new 25 cm^3^ flask and incubated at 22 °C ± 2 °C or at 37 °C ± 2 °C. This time point corresponded to the T_0_ time point of the assay. Each condition was performed for three independent replicates and repeated three times (n = 9), with the exception of the co-culture with strain Philadelphia that was repeated four times at 22 °C (n = 15).

### 4.6. L. pneumophila and Amoeba Quantifications in Co-Culture Assays from T_0_ to T_0_ + 96 h 

Occurring at T_0_, T_0_ + 24 h, T_0_ + 48 h, T_0_ + 72 h, and T_0_ + 96 h, a washing step was performed. The culture supernatant was removed from each flask and replaced by 10 mL of sterile SCYEM. This step was intended to remove extracellular *L. pneumophila* to allow for the detection of only intracellular bacteria. Each flask was gently shaken 10 times and an aliquot of 1 mL was sampled. Quantification of amoeba populations was performed using 0.1 mL of each aliquot utilizing a haemocytometer cell counting chamber method with Trypan blue. The remaining 0.9 mL were treated with Triton™ X-100 [31] at 0.02% v/v (final concentration) for 2 min to lyse amoebas and to recover the internal *L. pneumophila*. The sample was then serially 10-fold diluted in SCYEM and plated on BCYE plates in triplicate, with the exception of the undiluted conditions that were spread onto five plates when the number of *L. pneumophila* was intended to decrease below the detection limit. BCYE plates were incubated at 36 °C, and CFU were counted after 5 days.

### 4.7. Microscopic Observations in Co-Culture with L. pneumophila Philadelphia at 37 °C 

Co-cultures of *L. pneumophila* Philadelphia using the three amoeba strains at 37 °C were sampled from running experiments and stained by the Gimenez technique [50,51] at T_0_, T_0_ + 48 h, and T_0_ + 96 h. Co-cultures (0.1 mL) were deposited onto glass slides by using a Shandon Cytospin 4 cytocentrifuge (Thermo Scientific, Illkirch-France) at 800× *g* for 10 min and then stained using the Gimenez technique. Briefly, each of the glass slides were stained with fuchsin solution for 3 min and washed with water. Then, the glass slides were stained with malachite green for 5–10 s and washed, and this step was repeated twice. Finally, the glass slides were allowed to dry at room temperature. 

The observations were performed using a LEICA DM 2500 LED microscope (Leica Microsystemes SAS, Nanterre-France) under an ×100 oil immersion objective.

### 4.8. Statistical Analyses

Statistical significance of co-culture studies was determined for 22 °C and 37 °C conditions through the use of analysis of variance (ANOVA) (Kruskal–Wallis test and multiple pair-wise comparison Dunn test).

## Figures and Tables

**Table 1:** Statistical analysis of the behaviour of the three amoeba strains in the presence of the three *Legionella* strains at 22 °C and 37 °C. Significant differences for *W. magna* C2c Maky are highlighted in yellow.

	22 °C					37 °C				
*L. pneumophila* Lens	T0	24 h	48 h	72 h	96 h	T0	24 h	48 h	72 h	96 h
**With *A. castellanii***	A	A	A	A	A	A	A	A	A	A
**With *W. magna* Z503**	A	A	A	A	A	AB	AB	A	A	A
**With *W. magna* C2c Maky**	A	B	B	B	B	A	B	B	C	B
***L. pneumophila* Paris**	T0	24 h	48 h	72 h	96 h	T0	24 h	48 h	72 h	96 h
**With *A. castellanii***	A	A	A	A	A	C	A	A	A	A
**With *W. magna* Z503**	A	A	A	A	A	B	A	B	B	B
**With *W. magna* C2c Maky**	A	B	B	B	B	A	B	C	C	C
***L. pneumophila* Philadelphia**	T0	24 h	48 h	72 h	96 h	T0	24 h	48 h	72 h	96 h
**With *A. castellanii***	A	A	A	AB	AB	AB	A	A	AB	AB
**With *W. magna* Z503**	A	A	A	A	A	B	B	B	A	A
**With *W. magna* C2c Maky**	A	A	A	B	B	A	A	B	C	C

**Table 2:** Preparation of the co-cultures.

Co-Culture	AWS ^1^ Volume	BWS ^2^ Volume
*L.p.* Philadelphia *+ W. magna* C2c Maky	10 mL AWS_C2C_	0.1 mL BWS_Phila_
*L.p.* Philadelphia *+ W. magna Z503*	10 mL AWS_Z503_	0.1 mL BWS_Phila_
*L.p.* Philadelphia *+ A. castellanii.*	10 mL AWS_AC_	0.1 mL BWS_Phila_
*L.p.* Paris *+ W. magna* C2c Maky	10 mL AWS_C2C_	0.1 mL BWS_Paris_
*L.p.* Paris *+ W. magna Z503*	10 mL AWS_Z503_	0.1 mL BWS_Paris_
*L.p.* Paris *+ A. castellanii.*	10 mL AWS_AC_	0.1 mL BWS_Paris_
*L.p.* Lens *+ W. magna* C2c Maky	10 mL AWS_C2C_	0.1 mL BWS_Lens_
*L.p.* Lens *+ W. magna Z503*	10 mL AWS_Z503_	0.1 mL BWS_Lens_
*L.p.* Lens *+ A. castellanii.*	10 mL AWS_AC_	0.1 mL BWS_Lens_
*Control L.p.* Philadelphia	10 mL SCYEM	0.1 mL BWS_Phila_
*Control L.p.* Paris	10 mL SCYEM	0.1 mL BWS_Paris_
*Control L.p.* Lens	10 mL SCYEM	0.1 mL BWS_Lens_
*Control W. magna* C2c Maky	10 mL AWS_C2C_	0 mL
*Control W. magna Z503*	10 mL AWS_Z503_	0 mL
*Control A. castellanii*	10 mL AWS_AC_	0 mL

^1^ AWS: Amoeba Working Solution at 3 × 10^5^ cells / mL; ^2^ BWS: Bacteria Working Solution at 3 × 10^7^ CFU / mL.
